# Towards sustainable community conservation in tropical savanna ecosystems: a management framework for ecotourism ventures in a changing environment

**DOI:** 10.1007/s10668-020-00772-4

**Published:** 2020-05-20

**Authors:** Boycen Kumira Mudzengi, Edson Gandiwa, Never Muboko, Chiedza Ngonidzashe Mutanga

**Affiliations:** 1 Department of Geography and Environmental Science, Great Zimbabwe University, Masvingo, Zimbabwe; 2 School of Wildlife, Ecology and Conservation, Chinhoyi University of Technology, Chinhoyi, Zimbabwe; 3 School of Hospitality and Tourism, Chinhoyi University of Technology, Chinhoyi, Zimbabwe

**Keywords:** CAMPFIRE, CBNRM, Conservation, Ecotourism, Environmental sustainability, Mahenye

## Abstract

The sustainability of ecotourism ventures under the Community Based Natural Resources Management in Zimbabwe is under stress due to environmental changes emanating from effects associated with socioeconomic factors, climate change and epidemic diseases. Using an in-depth analysis of the Mahenye ecotourism venture from the year 1982 to 2020 as a case study, this study sought to propose a management framework for ecotourism ventures in a changing environment by examining the sustainability of community conservation initiatives in Zimbabwe. Research methods included expert opinion from two natural resources governance academics, desktop research and authors’ experiences about Mahenye ecotourism venture. Results indicated that the Mahenye ecotourism venture has faced significant challenges but has been resilient to withstand the shocks such as population increase, exclusion of youths and women, climate change, hyperinflation, donor fatigue, reduced international ecotourist visitation and international hunting bans emanating from socioeconomic and political environmental changes. These shocks have a negative effect on the main elements of an ecotourism venture such as the wildlife resources, amenities, attraction, accessibility, management system, marketing, beneficiaries and linkages. The management framework highlights the interventions that can be made to enable ecotourism ventures in changing environments to remain sustainable. The interventions are promoting strong community cohesion, developing sustainable self-funding mechanisms, promoting multiple sources of income, carrying targeted environmental education programs, capacity building in managing ecotourism in periods of hyperinflation, improved marketing and offering a unique experience, promoting climate smart ecotourism, promoting domestic ecotourism visits, implementing effective feedback systems among stakeholders to decrease uncertainties and lobbying to have hunting bans removed.

## Introduction

The environmental sustainability of Community Based Natural Resources Management (CBNRM) programs modeled in the form of the Communal Areas Management Programme for Indigenous Resources (CAMPFIRE) in Zimbabwe are under immense stress due to pressures induced by local socioeconomic changes (Mudzengi and Chiutsi 2014; Machena et al. 2017), effects of climate change (Murphree 2001; Mudzengi et al. 2020) and emergence of epidemic and contagious infections such as the novel coronavirus disease (COVID-19). Some of the CAMPFIRE projects such as the Mahenye ecotourism project in Chipinge District, Zimbabwe, faced significant challenges in the face of shocks resulting from a changing socioeconomic and biophysical environment in the country. The socioeconomic environment deteriorated after the year 2000 following the fast-track land reform, characterized by hyperinflation, corruption, widespread political violence and intolerance, low productivity in agriculture and industry, high rates of unemployment, negative media framing and isolation by the international core and donor countries (Balint and Mashinya 2006; Mudzengi and Chiutsi 2014; Gandiwa et al. 2014a; Machena et al. 2017), and in 2020, there are shocks emanating from the COVID-19 pandemic.

The socioeconomic challenges that later faced the Mahenye ecotourism project after 2000 are also associated with elite capture, poor management, nepotism, intimidation, ineffectiveness of local participatory processes, withdrawal of donor funding, removal of external capacity building support, and lower revenues due to poor macro-economic environment (Balint and Mashinya 2006; Machena et al. 2017; Tchakatumba et al. 2019; Mudzengi et al. 2020). This is in stark contrast to the Mahenye ecotourism success stories reported by Murphree (2001) before the year 2000. In fact, the success stories of ecotourism were not just confined to Mahenye ecotourism project but were widely reported in Chiredzi, Beitbridge, Hurungwe, Gokwe North, Gokwe South, Muzarabani and Rushinga Districts (Bond 2001; Murombedzi 2001; Taylor 2001). However, wildlife conservation in Zimbabwe after the year 2000 should also be understood in terms of the spillover effect in media framing whereby as the country was facing political crisis and economic collapse it was also felt that the negative conceptions of wildlife conservation status were associated with media reporting (Gandiwa et al. 2014a). This spillover effect seemed to have influenced policy makers leading to important international donors and wildlife conservation organizations withdrawing their support and funding to CBNRM initiatives in the country (Gandiwa et al. 2014a; Machena et al. 2017). Hence, the current CBNRM model is under pressure to redeem itself and breathe life to most ecotourism projects given the changing local socioeconomic environment.

The current challenges undermine the precepts upon which the CBNRM programs are founded. CBNRM represents a shift from a protected area model or fortress conservation toward more active local participation in natural resources conservation by giving local communities a great stake in conservation (Boonzaaier 2012). This approach resulted in the development and adoption of the CBNRM model in Eastern and Southern Africa (Riyoh 1995; Barnes et al. 1999; Barrow and Murphree 2001; Blaikie 2006; Machena et al. 2017). In Sub-Saharan Africa, CBNRM has become the dominant development discourse for environmental resource management processes (Nelson and Agrawal 2008; Poteete 2009). CBNRM represents a shift from a top-down approach to resource management toward the bottom-up approach which emphasizes the need for adequate consultation with local communities and stakeholder participation thereby supports the Subsidiarity Principle, whereby resource management decisions should be made at the lowest possible level (Alpert 1996; Boonzaaier 2012; Machena et al. 2017).

Thus, under CBNRM, there is mass participation and democratization of resource management. This contrasts with the fortress protection approach which involved centralized resource management (Blaikie 2006; Nelson and Agrawal 2008; Poteete 2009; Boonzaaier 2012). Centralized resource management usually results in fragmented resource management, overlap of responsibilities among resource governing institutions leading to conflicts and duplication of duties. Under centralization reactive approach (firefighting) to environmental problems is evident instead of proactiveness. Furthermore, people usually resist environmental regulations imposed from a central authority, especially where they feel that their interests were trampled upon. Centralized resource management and its fortress protection approach also often lead to negative protected area staff–local community relationships which do not enhance wildlife conservation (Mutanga et al. 2015; Mutanga et al 2017b). Thus, under CBNRM there is movement toward integrated resource management as opposed to piecemeal approach which characterized the protected area model (Mohamed-Katerere and Chenje 2002; Blaikie 2006; Poteete 2009; Boonzaaier 2012).

CBNRM also ensures that local communities benefit from environmental resources found in their area and hopes that communities develop a sense of stewardship over the resources leading to environmental sustainability. Community ecotourism is a subtype of CBNRM and involves having fun while supporting the protection of the natural and cultural resources. It also involves maintaining a low visitor impact and providing the local community with socioeconomic benefits (Cetin and Sevik 2016). Ecotourism is also supportive of pro-poor tourism initiatives whereby poor and often marginalized local communities around environmentally endowed protected areas can accrue socioeconomic benefits through tourism (Goodwin 2009). Pro-poor tourism and conservation initiatives can actually be the panacea for local communities’ development and environmental sustainability (Scheyvens 2007; Chiutsi and Mudzengi 2012; Snyman 2017). Given the vast benefits of ecotourism projects it is of interest to explore factors which can lead to the failure of these projects and also proffer solutions that ensure their sustainability.

This study’s objectives were to: (1) explore the factors related to the resilience of ecotourism at Mahenye in the face of shocks emanating from a changing operating environment, (2) examine the factors associated with the decline of ecotourism at Mahenye, and (3) analyze the lessons learnt from the decline of ecotourism at Mahenye. The results from the above three objectives were then used to propose a management framework for ecotourism ventures in changing environments. Information generated by this study could inform environmental policy makers and planners on ways of ensuring the continued sustainability of community-based ecotourism projects in a changing operating environment.

## Methods

### Study area

**Fig. 1:**
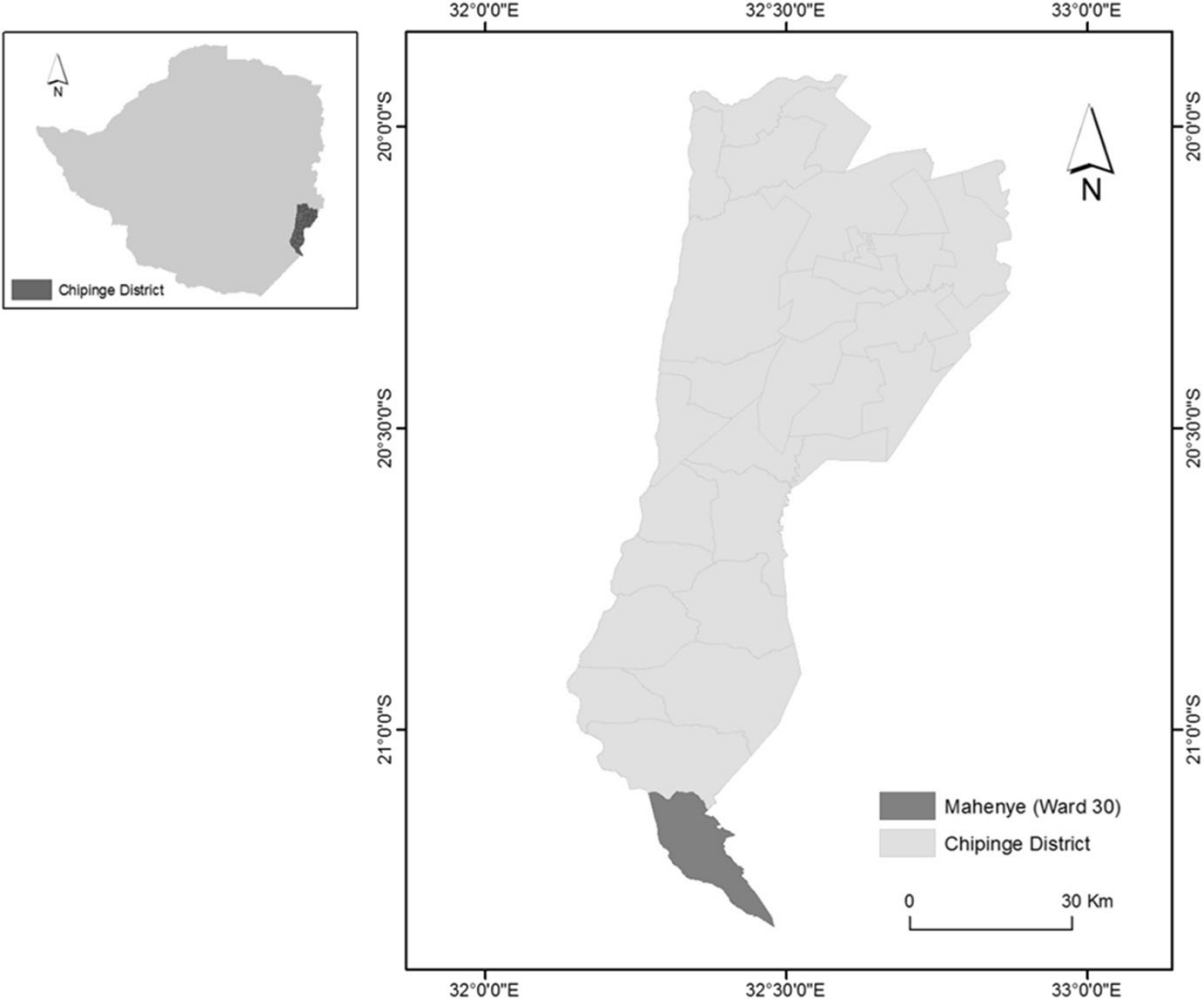
Location of Mahenye in southeast Zimbabwe. *Source* Authors

The Mahenye ecotourism venture started as a community public partnership between the local Shangaan-speaking peoples and the then Department of National Parks and Wildlife Management, now Zimbabwe Parks and Wildlife Management Authority in 1982. This arrangement was formally endorsed when the central government granted appropriate authority over wildlife to Chipinge Rural District Council in 1991 (Murphree 2001). Mahenye ecotourism then became a joint venture agreement between the local peoples and African Sun Limited (formerly Zimbabwe Sun Limited), which owns a chain of hotels in Zimbabwe in 1996 (Mudzengi and Chiutsi 2014). The Mahenye case study was chosen as it has been resilient in the face of significant challenges emanating from a changing socioeconomic environment in Zimbabwe (Gandiwa 2011; Wild Programme 2015; Machena et al. 2017; Tchakatumba et al. 2019) and climate change effects (Murphree 2001; Mudzengi et al. 2020). Mahenye lies at the extreme southern end of Chipinge District (southeast Zimbabwe), covering about 210 km^2^ in the Ndowoyo Communal Land (Fig. 1). Average rainfall is low (450–500 mm per annum) and supports little rain fed crop cultivation making ecotourism an important non-agricultural source of livelihood (Murphree 2001). Mahenye is also characterized by a tropical savanna ecosystem experiencing alternating dry cool winters and wet hot summers. The average temperatures for winter and summer are 27.5 °C and 32.5 °C, respectively (Chenje et al. 1998). Crop cultivation during the dry season is not viable without irrigation. The study area is covered by mixed mopane and *Combretum* woodland but a dense riverine forest is found along the Save River supporting a broad range of floral and avian species, some of them rare in Zimbabwe (Murphree 2001). Up until the official establishment of the ecotourism venture in 1982, the Shangaan used to illegally hunt wild animals extensively from the neighboring Gonarezhou National Park which was established using the protected area model, from which many of them were evicted when a game reserve was created in 1968 (Murphree 2001). The villagers poached as means of survival (Murphree 2001). The villagers also poached in the hope that if they killed wildlife no more tourists would come and the Gonarezhou National Park which had denied them access to natural resources which they exploited before its establishment would be eventually closed (Scheyvens 2000). However, with the establishment of the ecotourism venture, incidences of poaching declined (Murphree 2001; Gandiwa 2011). Strengthened law enforcement also ensured that illegal hunting did not increase significantly in the post-2000 period characterized by socioeconomic and political instability (Gandiwa et al. 2013a; Gandiwa 2014).

### Research approach

We approached the study from a qualitative perspective basing the study largely on review of relevant literature. The review of literature is supplemented by data from expert opinion of 2 academics at the University of Zimbabwe’s Centre for Applied Social Sciences and authors’ experiences about the study area which spans for 15 years undertaking research work. The study embarked on an in-depth analysis of the Mahenye ecotourism venture using the case study approach. A case study is a research approach involving an in-depth, multi-faceted and detailed examination of a subject of study, as well as its related contextual conditions (Yin 1994). The study was based largely on a review of literature relating to the factors that have led to the Mahenye ecotourism venture facing significant challenges given that ecotourism is one of the fastest-growing segments of the tourism industry (Anup et al. 2015; Das and Chatterjee 2015; Hultman et al. 2015) with great potential to contribute toward the conservation of biophysical resources (Kapil and Pierre 2017) and local community development (Scheyvens 2000). The reviewed literature also relate to the performance and resilience of the Mahenye ecotourism project from the year 1982 to 2020, a timeframe covering the period before and after the emergence of socioeconomic and political environmental changes in Zimbabwe from the year 2000. The reviewed literature also relate to the CBNRM model and ecotourism sustainability in Zimbabwe in particular and Sub-Saharan Africa, Asia, Central America, South America, the Caribbean, and Europe in general. The review of the literature was approached from a holistic, historical and comparative perspective (Gandiwa et al. 2014a) which enabled us to understand the factors associated with CBNRM sustainability in Zimbabwe. The holistic perspective allowed us to examine the broader issues related to CBNRM sustainability since it helps identify the connections between and interactions of various factors. The historical and comparative perspective enabled us to analyze and compare the factors related to CBNRM sustainability in the country before and after the emergence of socioeconomic and political environmental changes. The two periods that were compared were the pre-2000 and post-2000 eras in Zimbabwe. The pre-2000 era was characterized by relative macro-economic and sociopolitical stability as compared to the post-2000 era in which the country is experiencing a negative macro-economic environment, low economic productivity, low investor confidence, high unemployment, and political intolerance.

### Data collection and analysis

Academic literature on CBNRM and ecotourism sustainability was collected from 89 peer reviewed journal articles, books, edited book chapters, statistical reports and academic theses from October 2017 to April 2020. These literature sources were selected as they discussed in detail the sustainability of ecotourism mainly covering tropical savanna countries in Sub-Saharan Africa, Asia, Central America, and the Caribbean. This points to the fact that our findings apply more to tropical savanna ecosystems with, however, some aspects still applicable to other parts of the world. Academic literature search engines, namely Google Scholar, Scopus and Web of Science were also used to retrieve relevant literature from the internet. The internet scholarly search engine inquiry was guided by inputting the following key words or phrases: “CBNRM”, “ecotourism”, “community conservation”, “community-led conservation in Africa”, “CAMPFIRE”, “CBNRM in Zimbabwe”, “CBNRM and environmental sustainability” and “ecotourism and environmental sustainability”. The literature, which contained the aforementioned key words or phrases in the abstract were included in the analysis list, while the rest of the literature were discarded. The study also used the authors’ knowledge about Mahenye having carried out research in the area from the year 2004 to 2019. Expert opinion was also obtained from 2 natural resources governance academics at University of Zimbabwe’s Centre for Applied Social Sciences, hereafter referred to as Expert 1 and Expert 2 in February 2017. The experts were also interviewed on the performance and resilience of the Mahenye ecotourism venture. Expert opinion was sought in order to corroborate data from the literature and authors’ field experiences.

The collected literature and data relating to the sustainability of CBNRM was analyzed using the qualitative interpretative approach (Bhatta 2014). This was done by noting and synthesizing factors related to the resilience of ecotourism at Mahenye in the face of shocks emanating from a changing operating environment. Data analysis also included identifying and examining factors associated with the decline of ecotourism in Zimbabwe. The lessons learnt from the decline of ecotourism at Mahenye were determined based on lessons mostly mentioned in the reviewed literature. Some of the lessons were also mentioned by experts. The information obtained from data analysis and authors’ field experiences was then used to propose a management framework for ecotourism ventures in changing operating environments. The management framework was adapted from the Sustainable Livelihoods Approach (SLA) (Scoones 1998; Morse and McNamara 2013). The SLA is a diagnostic tool that provides a framework for understanding and improving the sustainability of livelihood in the face of biophysical and socioeconomic shocks. According to the SLA, a livelihood is sustainable when it can cope with and recover from stresses and shocks and maintain or enhance its capabilities and assets both now and in the future, while not undermining the natural resource base (Morse and McNamara 2013).

## Results and discussion

### Factors related to the resilience of ecotourism at Mahenye

The factors related to the resilience of ecotourism at Mahenye in the face of adversity are resource richness, favorable climate that does not have extreme heat or cold, intra-communal cohesiveness, enlightened private sector involvement, compensation to local communities for protecting natural resources, specific rules regulating access and no access to resources, specific roles of traditional leadership, effective conflict management strategies, commitment of socially dedicated individuals in positions of leadership (social energy), existence of multiple sources of income and environmental education programs to promote community environmental awareness and commitment to conservation. This was noted by Murphree (2001), Gandiwa et al. (2013a), Wild Programme (2015), Mudzengi et al. (2020), and the 2 Experts interviewed. The significance of these factors for ecotourism sustainability was also discussed in Mexico (Foucat 2002; Monteros 2002), Panama and Costa Rica (Lieberknecht et al. 1999; Cusack and Dixon 2006), Chile (Pilquimán-Vera et al. 2020); the Caribbean (Slinger 2000), and Turkey (Cetin 2015; Cetin et al. 2018).

Mahenye is regarded as a prime ecotourism project area as it had one of the highest revenues of all the areas participating in the CAMPFIRE in the 1990s (Murphree 2001). Mahenye has a rich biophysical resource endowment which is adequate to support an ecotourism enterprise yielding significant inputs to community infrastructure and annual revenues (Murphree 2001; Gandiwa 2011; Wild Programme 2015). Further, being based on both consumptive and non-consumptive ecotourism, the revenue earning potential of the Mahenye venture is significant and diversified (Murphree 2001; Wild Programme 2015). The Mahenye community also retains strong tradition and culture which is an additional asset to ecotourism development in the area. The tradition and culture of the Mahenye community has remained strong as the Shangaan are ethnically discrete within the Chipinge District. All the other wards of Chipinge District comprise primarily Shona-speaking Ndau peoples (Murphree 2001). Thus, Mahenye is characterized by discreteness and isolation. Traditional law also prohibits deforestation at Mahenye and these laws are enforced by the traditional leadership (Wild Programme 2015). Community cohesion was also important in ensuring the resilience of the Mahenye ecotourism venture (Murphree 2001; Gandiwa et al. 2013b). Community organization and cohesion is important for raising awareness about the benefits of ecotourism, equitable sharing of benefits and costs, information dissemination and conflict management (Cusack and Dixon 2006; Gandiwa et al. 2013b).

The enlightened partnership and linkage of the Mahenye ecotourism project with a private sector tourism company also led to improved road transport provision, electricity connections to the local clinic, school, grinding mill and the police post, telephone connections to the community center, water reticulation being extended to the village centre, provision of livestock watering points and employment provision (Murphree 2001; Mutanga et al. 2017b). According to Expert 1 conflict management strategies were effective from the initiation of the project up to the year 2000 in ensuring fair compensation to local communities for protecting natural resources and regulating access to resources. The expert also argued that the resilience of ecotourism at Mahenye was due to the existence of multiple sources of income that ensured that the venture was not abandoned at the first tourism slump. The Mahenye community is also involved in selling agricultural products as well as traditional handicrafts and traditional brews made from natural resources such as palm trees. The Mahenye CAMPFIRE committee also ensured that environmental education programs were provided to the community (Gandiwa 2011). The programs raised community environmental awareness and ensured local people were committed to make the ecotourism venture succeed. The comfortable climatic conditions at Mahenye also ensure ecotourists feel relaxed and satisfied. The benefits of bioclimatic comfort in ecotourism have been detailed by Cetin (2015) and Cetin et al. (2018). Further, individuals like the local chief, councillors, members of the Wildlife Committee, Clive Stockil, a local rancher and safari operator, and the local school headmaster were committed to see the ecotourism venture succeed (Murphree 2001).

The Mahenye ecotourism initiative was very positive in terms of promoting socioeconomic development in a marginalized communal area, encouraging sustainable use of natural resources and enhancing the control of local people over development in the surrounding areas from its inception in the year 1982 to 2004. The positive outcomes of the initiative during this time included more income and improved food security to the local community and more sustainable use of the natural resource base (Murphree 2001). The initiative then faced significant challenges from the year 2004. These significant challenges included low income accruing to the project due to withdrawal of funding by international donors in 2003 and reduced ecotourism visitation numbers (Gandiwa et al. 2014a). These challenges were significant as they threatened the financial viability of the project as ecotourism has to achieve economic, sociocultural, and ecological sustainability (Chiutsi et al. 2011; Mudzengi and Chiutsi 2014; Machena et al. 2017). These three pillars of sustainable development are interlinked and the failure of one threatens the viability of the other two. From the year 2004, the poor macro-economic environment in Zimbabwe emanating from the unintended negative effects of the fast-track land reforms which began in 2000 such as poor relations with Western nations, withdrawal of international conservation agencies from the country and reduced international tourist arrivals into the country (Bond and Manyanya 2002; Zimbabwe Tourism Authority 2008; Chiutsi et al. 2011) also started to take its toll on the project and on community social cohesion (Balint and Mashinya 2006; Mudzengi and Chiutsi 2014).

However, the Mahenye venture continued to run in the face of these challenges showing its resilience. Currently efforts are being made to enhance the Mahenye ecotourism program and restore it to its initial glory through the ongoing development of the Jamanda Community Conservancy and Development Trust which is being supported by the Wildlife in Livelihood Development (WILD) Programme (Wild Programme 2015). The Jamanda Community Conservancy and Development Trust incorporates the traditional leadership (Chief Mahenye and Headmen) and elected community representatives with whom other WILD stakeholders and Chilo Lodge work closely toward establishment of the Jamanda Wilderness Area which is envisaged to develop into a communal game park. These envisaged developments have great comparisons with Namibian community conservation initiatives (Machena et al. 2017).

The Jamanda Community Conservancy and Development Trust represents a further step in devolution of user rights over biophysical resources from the Chipinge Rural District Council to the Mahenye community. This will be done by giving the Mahenye community a more central role in management and decision-making in conservation-based enterprises in their area such as the envisaged creation of a communal game park. These ongoing innovative attempts of creating a community conservancy and development trust is expected to enable the Mahenye venture to improve its wildlife resource base thus, giving it more impetus. Further, being based on a non-consumptive ecotourism model the conservancy denotes a shift away from the trophy hunting model upon which CAMPFIRE has become somewhat overly dependent (Wild Programme 2015). However, according to the 2 Experts devolving user rights to the Mahenye community under the current land tenure system in Zimbabwe is problematic as the community is not a legal entity. The innovative attempt of creating a communal game park is also being hampered by lack of financial and technical resources to hire and train game scouts. Lack of financial and technical resources was also reported as being experienced in Botswana under the Natural Resource Management Programme (NRMP) (Machena et al. 2017).

The resilience of the Mahenye CBNRM project has some similarities with the experiences of Namibian community-led conservation initiatives under the Living in a Finite Environment (LIFE) project (Riyoh 1995; Jones and Murphree 2001). Under the LIFE project local communities work together with conservation agencies, international donors and private sector business players to enhance ecotourism development (Murphree 2001; Jones and Murphree 2001; Boudreaux 2010; Boudreaux and Nelson 2011; Machena et al. 2017). The initiation and resilience of the Mahenye project compares well with the experiences of the CBNRM initiative in the northwestern Kunene region of Namibia (Murphree 2001; Jones 2001; Boudreaux 2010). The success of both initiatives demonstrates the importance of the insights, ingenuity and commitment of dedicated individuals in positions of influence to the development and sustainability of CBNRM projects (Murphree 2001; Boudreaux 2010; Boudreaux and Nelson 2011; Machena et al. 2017). Namibian community-led conservation initiatives are largely regarded as more progressive and robust than others in Africa as they have succeeded in increasing the income and human capital of rural Namibians, strengthening local governance structures and leading to a recovery of wildlife species (Boudreaux 2010; Boudreaux and Nelson 2011; Machena et al. 2017).

### Factors associated with the decline of ecotourism at Mahenye

The factors that have contributed to the decline of ecotourism at Mahenye include elite capture, poor management, nepotism, intimidation, ineffectiveness of local participatory processes, withdrawal of donor funding, removal of external capacity building support and lower revenues due to a poor macro-economic environment in the country (Balint and Mashinya 2006; Mudzengi and Chiutsi 2014). The sustainability of CAMPFIRE came under increasing pressure after the year 2000 when Zimbabwe suffered a debilitating social, political and economic crisis following fast-track land reforms (Balint and Mashinya 2006; Mudzengi and Chiutsi 2014). This was worsened by the failure to implement effective feedback systems among stakeholders to decrease uncertainties resulting from the crisis according to Expert 2. The land reforms had a largely unintended negative backlash on the economy and society as it became the basis for international isolation of Zimbabwe by international core donor and trading states of Western Europe and North America who did not support the reforms, low investor confidence, low Foreign Direct Investment inflows, low productivity in agriculture and industry, high inflation, expensive and uncompetitive ecotourism packages and unemployment (Bond and Manyanya 2002; Mudzengi and Chiutsi 2014). CAMPFIRE revenues experienced a very large decline in the years 2004 and 2005 as a result of fatigue by many international donor agencies that withdrew their funding in 2003 (Gandiwa et al. 2014a). International relations with Western nations deteriorated, international tourist arrivals declined sharply, foreign earnings greatly constricted, foreign direct and portfolio investment in tourism, in particular, and other socioeconomic sectors dried up, international airlines withdrew, inflation rose, unemployment and underemployment increased and the Gross Domestic Product experienced negative growth (Bond and Manyanya 2002; Ferreira 2004; Balint and Mashinya 2006; Van Ameron 2006; Chiutsi et al. 2011; Mudzengi and Chiutsi 2014).

However, the income-generating potential of sport hunting was least affected by the crisis resulting in CAMPFIRE communities continuing to be resilient and accrue some revenue (Lindsey et al. 2007; Gandiwa et al. 2013a). Nevertheless, from 2004 the CAMPFIRE revenues were now lower than before meaning a further reduction in benefits to local communities (Gandiwa et al. 2013a). This was worsened by the fact that political and economic elites were less willing to further devolve access and control of wildlife resources from the Rural District Council level (Muboko and Murindagomo 2014). Fewer benefits meant that local communities were less willing to give up crop cultivation and grazing land for wildlife conservation as argued by Murindagomo (1998).

Hunting bans also have a negative effect on ecotourism revenues. For example, the ivory trading ban under the Convention on International Trade in Endangered Species of Wild Fauna and Flora (CITES) in the year 1989 meant that ecotourism communities were deprived of their major source of livelihood (Chenje et al. 1998; Gandiwa et al. 2014a). However, in 1999, the first international ivory sale after the ban was allowed after intense lobbying by Botswana, Namibia and Zimbabwe at a meeting in Harare in June 1997 (Chenje et al. 1998; Gandiwa et al. 2014a). At the Harare meeting the three countries successfully convinced the international community to downlist the African elephant (*Loxodonta africana*) from Appendix I to II, allowing limited trade in ivory. The ivory ban was again restored in 2001 at a CITES meeting in Nairobi, Kenya, and then another ivory sale was approved in 2008 at a meeting in Geneva, Switzerland (Gandiwa et al. 2014a). According to Expert 2 in the year 2014, the USA also banned its citizens from transporting and bringing into the country ivory from Tanzania and Zimbabwe. As a result of this ban, CAMPFIRE income dropped to US$2.1 million in 2014 then to US$1.2 million in 2015 as compared to US$2.3 million in 2013 (Machena et al. 2017). According to Expert 2 this ban was removed in 2016 following some lobbying. These hunting bans give more impetus to the development of other non-consumptive ecotourism activities such as photographic safari and animal viewing which are currently being explored at Mahenye by way of creating a communal game park (Gandiwa et al. 2014a; Wild Programme 2015). This development is expected to enhance the ecotourism visitor experiences in Mahenye (Wild Programme 2015).

The post-2000 socioeconomic problems have a complex history and are shaped mainly by the links between the residual effects of British colonial rule and struggles over the country’s future political and economic destiny (Balint and Mashinya 2006). The problems have also been worsened by economic and political instability (Murphree 2001; Mudzengi and Chiutsi 2014). Further, starting from the year 2000 the government implemented an accelerated land redistribution program that in some areas degenerated into chaotic and often violent takeover of former European settler owned commercial farms and ranches, and even some sections of government-owned national game parks and protected areas such as Gonarezhou (Bond and Manyanya 2002; Chaumba et al. 2003; Wolmer et al. 2004; Chigonda 2018). As a result of these developments, some international core donor states in the rich Global North such as European Union countries and the USA imposed sanctions on the country (Mudzengi and Chiutsi 2014; Chiutsi et al. 2011). Also some foreign companies and donors left the country (Bond and Manyanya 2002).

Ecotourism at Mahenye also declined due its failure to actively involve women and the youth in natural resources management (Gandiwa et al. 2014b). This failure is also evident in South Africa (Machena et al. 2017). Women utilize natural resources more than men as they are in constant contact with soil, water and forest resources through crop cultivation, fetching water and firewood hence there is need to ensure their increased participation in ecotourism programs, especially at the decision-making level (Dankelman and Davidson 1988; Mudzengi et al. 2013; Mashapa et al. 2019, 2020). The youth also regard natural resources management endeavors under ecotourism as poorly rewarding financially and are less willing to reside in socioeconomically disadvantaged and often marginalized communal lands thereby migrate to urban economic core areas (Gandiwa et al. 2014b). Socioeconomic deprivation in the communal areas has also been worsened by the poor macro-economic environment in the country.

However, before the year 2000 CAMPFIRE was also facing challenges emanating from increased competition for land for agricultural (crop farming and livestock grazing) and wildlife uses. This was due to human population increase in CAMPFIRE wards as a result of natural increase and in-migration. Natural population increase was due to better provision of health services (Murombedzi 2001; Chazireni 2018). In-migration into CAMPFIRE wards was due to low population densities in these areas and people who were moving into rural areas after being retrenched from formal employment in urban areas (Murombedzi 2001). The increases in human population in CAMPFIRE areas reduced the dividends per capita from CAMPFIRE earnings especially from trophy hunting (Murombedzi 2001). Further, there were challenges emanating from climate change as the trophy hunting quota was not achieved during drought years resulting in lower revenues accruing to communities, for example, from trophy elephant (Murphree 2001). Gandiwa (2014) also note that drought has a negative effect on some large herbivore populations in tropical savanna ecosystems. These challenges did not, however, result in the degeneration of CAMPFIRE projects and the program was able to withstand these stresses throughout the 1990 s (Murphree 2001). This was partly because the program was still receiving external support from international donors and conservation agencies and partly because the socioeconomic and political environment in the country was still conducive for tourism and business (Mudzengi and Chiutsi 2014; Gandiwa et al. 2014a). Flooding due to climate change also led to the extensive damage of roads and the Mahenye Safari Lodge in 2008 (Mudzengi et al. 2020).

### Lessons learnt from the decline of ecotourism at Mahenye

One important lesson to be learnt from the decline of the Mahenye ecotourism venture is that local communities on their own without technical support and business marketing capacitation from established tourism enterprises and conservation agencies cannot maintain the overall sustainability of these local community-managed projects. This is also supported by (Balint and Mashinya 2006; Machena et al. 2017) who note that local participatory decision-making and resource-governing institutions are fragile and therefore require continuous external support and capacity building from established conservation agencies in managing ecotourism initiatives. However, there is also need for sustainable self-funding as ecotourism ventures have to limit their dependence on external support.

In addition, another important lesson is that just relying on international tourists is a weakness of most community-based ecotourism ventures especially in southern Africa such as the Mahenye case. There is therefore need to promote domestic ecotourism visitation by Zimbabweans not just international tourism (Mutanga et al. 2017a). The Mahenye case demonstrated the significance of the market segment from rich international core states of the Global North mainly in Western Europe and North America in sustaining the ecotourism venture. Ecotourist arrivals are important in ensuring the economic sustainability of ecotourism projects. Zimbabwe’s poor relations with the Western tourist source countries starting from the year 2000 have led to the broader international market boycotting the country as a tourist destination. The country has largely been seen as an unsafe destination leading to tourist arrivals declining sharply. Thus, the possession of abundant attractive natural resources alone without a good security image and favorable relations with the international core tourist states of the Global North is not adequate to ensure the sustainability of ecotourism initiatives that rely on international tourism such as Mahenye. Indeed at the peak of the socioeconomic instability in the country major tourist source nations such as the USA, Britain, Australia and Japan issued travel warnings to their nationals against traveling to Zimbabwe (Zimbabwe Tourism Authority 2008).

These travel warnings were lifted with the somewhat stabilizing of the situation following the formation of the Government of National Unity which governed from the year 2009 to 2013 and tourist arrivals started to increase as evidenced by a 3% increase to 2 017 264 tourist arrivals in 2009 as compared to 2008 (Zimbabwe Tourism Authority 2009). Tourist arrivals further increased to 2,239,165 in 2010 and 2,423,280 in 2011 (Zimbabwe Tourism Authority 2011). Tourist arrivals declined to 1,832,570 in 2013 (Zimbabwe Tourism Authority 2013) as it was an election year and elections are associated with political instability in the country. The United Nations World Tourism Organization assembly which Zimbabwe co-hosted with Zambia in August 2013 also presented an opportunity to work on the unfavorable image the international community has on the country and further improve tourist arrivals. With the recent political developments in Zimbabwe which occurred in November 2017 and the subsequent harmonized national elections in July 2018 which saw the coming in of a new administration in Harare that shows keenness to improve relations with all the nations of the world, the number of tourist arrivals into Zimbabwe has been increasing (Parliament Budget Office 2018; Chigonda 2018).

At the peak of the Mahenye ecotourism venture due to increased cash flow in the community from ecotourism the purchasing power for agricultural inputs increased leading to increased demand for cultivation and grazing land. The Chilo Lodge and Mahenye Safari Lodge also disturbed livestock grazing and watering patterns. As a result, there was increased competition and conflicting interests for land to use for agriculture and wildlife conservation (Mudzengi and Chiutsi 2014). However, these conflicting interests did not lead to the collapse of the venture thus, showing its resilience. Human-wildlife conflicts have also been witnessed in ecotourism ventures in South Africa (Boonzaaier 2012), Namibia (Boudreaux 2010; Boudreaux and Nelson 2011; Machena et al. 2017) and Botswana (Mbaiwa 2011; Mbaiwa and Stronza 2011; Lenao 2013; Stone and Nyaupane 2017).

Social and cultural changes induced by ecotourism ventures may also threaten the sustainability of such initiatives. At the peak of the Mahenye ecotourism venture employment was highly skewed in favor of men at the expense of women and such gender disparity may threaten the overall sustainability of the project (Murphree 2001). This is so as fewer benefits for women would result in them being less inclined to support the ecotourism initiatives, yet they are the ones in constant contact with natural resources that are important for sustaining ecotourism. Furthermore, local employment is skewed toward lower-paid categories and this can lead to tensions between the locals and workers from outside Mahenye who are employed in higher-income bracket jobs (Murphree 2001). These tensions are associated with hatred and animosity between locals and workers from outside Mahenye. Other negative outcomes are that the Chilo Lodge and Mahenye Safari Lodge bring in outsiders and cases of petty thievery have increased. The behavior and forms of dress of lodge visitors are also regarded by some as inappropriate (Murphree 2001).

Further, there are complaints that the lodges have restricted community access to bathing and fishing points on the Save River (Murphree 2001). There are also worries that wage structures giving younger workers higher salaries than their elders may upset traditional hierarchies of respect. Ecotourism can also limit the access of traditional healers to medicinal plants (Boonzaaier 2012). Ecotourism is also often associated with increased incidences of prostitution and commodification and commercialization of culture and biophysical resources (Stone and Nyaupane 2017). Thus, more plant species may have to be cut to make cultural artefacts to sell to tourists (Stone and Nyaupane 2017). These costs can increase conflicts and tensions within a community engaged in ecotourism (Nyaupane and Poudel 2011; Bramwell and Lane 2013; Saarinen and Lenao 2014; Stone and Nyaupane 2016, 2017). Thus, such costs may have a negative effect on the sustainability of ecotourism initiatives in Zimbabwe.

Furthermore, ecotourism ventures such as Mahenye should not be guided by the neoliberal development approach if they are to be beneficial to local communities in the Global South. The main argument underlying neoliberalism is that the most effective way of achieving economic growth is to adopt a free market system in which all goods and services are produced and distributed by the private sector and the role of government is merely to provide a conducive environment for private investment (Konadu-Agyemang 2000; Telfer and Sharpley 2008). Neoliberalism postulates that spatial and socioeconomic inequalities are inherently inevitable and even necessary when economies are growing (Konadu-Agyemang 2000). Neoliberalism is also not supportive of pro-poor tourism in Sub-Saharan Africa in particular and the Global South in general (Scheyvens 2007). To some extent, neoliberalism influenced the development of ecotourism at Mahenye as it was the approach followed by the Zimbabwean Government when the venture was formally endorsed. The Zimbabwean Government launched the Economic Structural Adjustment Programme which had the support of the Bretton Woods Institutions in 1991. However, neoliberalism was abandoned after 2000 due to poor relations with the West. Further, some aspects of these neoliberal development approaches are not supportive of communal ownership of resources (Bhatta 2014; Regmi and Walter 2017). Thus, community-based ecotourism projects in Sub-Saharan Africa should shift from being guided by neoliberalism and the Eurocentric and capitalist oriented modernization approach and be more locally controlled in order to be sustainable. Such thinking is also supported by authors like Bhatta 2014 and Regmi and Walter 2017. These authors argue that for ecotourism to be sustainable in Nepal and other countries of the Global South it should be more locally controlled instead of being dominated by large foreign companies and local elites.

It can therefore be noted that for successful community-based ecotourism ventures, the local people should have a thicker bundle of property rights that enable them to have more opportunities to benefit from their biophysical resources. This will in turn ensure that local people have more control over socioeconomic development in their area and are not exploited by powerful local political and economic elites and international capital. Devolution to the community level also ensures that local leadership which includes chiefs, village headmen and community conservation management committee members are more accountable to the local people than to international donors, conservation agencies, and private business ecotourism players. This is important as it is common for private business to seek to pay very low rates for access to community wildlife resources, and concentrate payments in the hands of local leadership elites who, in turn become rentiers of the community’s resources and sometimes cheat poor villagers in both blatant and sophisticated ways (Hulme and Murphree 2001a, b). These corrupt tendencies by local elites were also observed by (Balint and Mashinya 2006) at Mahenye.

### Management framework for ecotourism ventures in changing environments

The adverse socioeconomic developments in the country after the year 2000 have resulted in ecotourism initiatives experiencing negative outcomes and significant challenges, and Mahenye has not been spared. Environmental changes induce shocks that affect the main elements of an ecotourism venture such as the wildlife resources, amenities, attraction, accessibility, management system, marketing, beneficiaries and linkages. Given such a scenario, it therefore becomes important to develop mechanisms that ensure that ecotourism initiatives are able to adapt to changing socioeconomic circumstances thereby becoming resilient in the face of shocks. This study argues that for ecotourism programs to be viable they have to be economically, socially and biophysically sustainable. Political stability is also critical for the success of ecotourism projects. Further, effective strategies need to be developed to respond to emerging shocks such as zoonotic diseases and pandemics as the COVID-19. These zoonotic diseases and pandemics have also increased calls for the banning of wildlife trade. This is so as the majority of these emerging infectious diseases originate in wildlife (Jones et al. 2008). These emerging shocks have a bearing on the broader ecotourism issues. The COVID-19 pandemic has resulted in lockdowns, travel restrictions and closure of ecotourism facilities and businesses. Developing management frameworks that ensure that ecotourism ventures remain resilient in the face of shocks induced by a changing local operating environment becomes imperative in tropical savanna ecosystems and other parts of the world. Our management framework for ecotourism ventures in changing environments (Table 1) shows environmental shocks effecting tourism and possible management interventions as well as livelihood outcomes.

**Table 1:** Proposed management framework for ecotourism ventures in changing environments. *Source* Authors

Ecotourism element	Shock	Effect	Management interventions	Livelihood outcomes
Beneficiaries Marketing Linkages	Reduced international ecotourist visitation	Reduced income from ecotourism	Promoting domestic ecotourism visits Promoting multiple sources of income Promoting favorable relations with the major international tourist source nations	More income to venture Increased community well-being Reduced vulnerability to shock
Beneficiaries Marketing Linkages	International hunting bans	Loss of major source of ecotourism income	Lobbying to have ban removed Diversification to non-consumptive ecotourism activities Promoting multiple sources of income	More income to venture Increased community well-being Improved supply of meat protein and food security More sustainable use of natural resource base Reduced vulnerability to shock
Beneficiaries Marketing Linkages	Exclusion of youth and women	Failure of youths and women to benefit from natural resources management Migration of youth to urban settlements	Promoting the involvement of youth and women in natural resources management Carrying targeted and effective environmental education programmes to raise awareness	Increased community well-being Reduced vulnerability to shock
Beneficiaries Wildlife resources	Human population increase	Competition for land for agricultural and wildlife uses Reduced dividends per capita from trophy hunting	Promoting effective and strong community organization and cohesion Promoting multiple sources of income Diversification to non-consumptive ecotourism activities	Reduced vulnerability to shock More sustainable use of natural resource base
Management system Beneficiaries Linkages	Donor fatigue	Decline in earnings Removal of external capacity building support Increased ineffectiveness of local participatory processes	Developing sustainable self-funding mechanisms Limiting dependence on donors Promoting multiple sources of income	Increased community well-being Reduced vulnerability to shock
Beneficiaries Management system Marketing Accessibility Amenities	Poor macro-economic environment in the country	Reduced ecotourist visitation Hyperinflation Withdrawal of international airlines from the country Failure to maintain roads and amenities Uncompetitive and expensive ecotourism packages Increased incidences of corruption Social disruption	Improved marketing and offering a unique ecotourism experience Implementing effective feedback systems among stakeholders to decrease uncertainties Capacity building in managing ecotourism in periods of hyperinflation Promoting sport hunting	Reduced vulnerability to shock
Beneficiaries Wildlife resources Marketing Linkages Accessibility Amenities Attraction	Climate change	Failure to achieve trophy hunting quota leading to lower revenues Damage of roads, bridges and lodges due to floods Biophysical environmental degradation due to drought	Promoting climate smart ecotourism Applying and lobbying for funds to mitigate climate change effects from international conservation agencies	Reduced vulnerability to shock Increased community well-being

## Conclusions

The sustainability of ecotourism initiatives in Zimbabwe is threatened by socioeconomic and biophysical environmental changes as well as emerging infectious diseases such as the COVID-19. These environmental changes in the country have induced shocks which require ecotourism initiatives to be more resilient to resist and survive as evidenced by the Mahenye case study. The Mahenye ecotourism venture is on the mend and is leading the race for enhanced CBNRM models in Zimbabwe. Given that ecotourism is one of the fastest-growing segments of the tourism industry there is need to revive the declining CBNRM initiatives in Zimbabwe to their early glory and ensure that they play an important role in socioeconomic and local community development as well as conserving biophysical environmental resources. Thus, the current CBNRM model and approach needs to be adapted and transformed to ensure that other ecotourism initiatives in the country and other parts of the world remain resilient, such as what the Mahenye venture has done, in the face of stresses and pressures resulting from environmental changes. To this end, the current CBNRM model and approach can be adapted to be more robust and resilient using our management framework for ecotourism ventures in changing environments. It is hoped that our management framework can be applied to other countries in the tropical savannas with similar biophysical environmental settings as Zimbabwe, in particular, and the rest of the world in general for the sustainability of ecotourism ventures in changing operating environments.
